# Foundation doctor preparedness for treating mental health conditions: results from a national survey

**DOI:** 10.1192/bjo.2021.392

**Published:** 2021-06-18

**Authors:** George Gillett, Owen Davis, Amarit Gill, Clare van Hamel

**Affiliations:** 1 IoPNN King's College London; 2 UK Foundation Programme Leadership Fellow & Foundation Year 2 Doctor, Oxford Foundation School; 3 UK Foundation Programme Leadership Fellow & Foundation Year 2 Doctor, Wales Foundation School; 4 Severn Foundation School Director & Clinical Advisor to UK Foundation Programme

## Abstract

**Aims:**

Previous research suggests the prevalence of mental health conditions among medical inpatients may be as high as 38%. Anecdotally, junior doctors report lacking the confidence, knowledge and skills to assess and treat patients with psychiatric conditions. Identifying this unmet need offers potential to improve standards of care and achieve parity of esteem between psychiatric and medical conditions within the general hospital. Aims:

To assess self-reported preparedness of newly-qualified Foundation Doctors to care for patients with acute or chronic psychiatric symptoms in comparison to physical health conditions.

**Method:**

In September of each year (2017, 2018, 2019), a survey was cascaded to all incoming Foundation Year 1 Doctors. For each respective year there were 1673, 961 & 1301 respondents. Respondents were asked to rate their agreement with statements on a Likert scale. Statements pertaining to mental health included “a) I am competent in acute mental health care provision, b) I am competent in chronic mental health care provision” and “I feel confident in prescribing the following drugs; c) drugs for mental health problems”. Comparison statements assessed confidence caring for medically unwell patients, performing practical procedures and prescribing drugs for physical health conditions.

**Result:**

Preparedness for acute and chronic mental health were lower than both physical health comparison items; preparedness to care for patients with critical illness (acute: r = 0.794, p < 0.001, chronic: r = 0.556, p < 0.001) and preparedness to perform practical procedures (acute: r = 0.724, p < 0.001, chronic: r = 0.433, p < 0.001).

Confidence prescribing mental health drugs was lower than all other comparison items (simple analgesia: r = 0.854, bronchodilators: r = 0.789, antimicrobials: r = 0.772, inhaled steroids: r = 0.720, intravenous fluids: r = 0.702, oral anti-diabetics: r = 0.611, anticoagulants: r = 0.515, narcotics: r = 0.514, insulin: r = 0.206; p < 0.001)

**Conclusion:**

These results identify a disparity in foundation doctors’ self-reported preparedness to treat acute and chronic mental health conditions and prescribe psychotropic medications, compared to a variety of physical health domains. To our knowledge this is the first large-scale study to empirically test a potential discrepancy between newly-qualified doctors’ preparedness to treat patients’ mental and physical health needs. Medical school education and foundation training may therefore present a fruitful opportunity to improve care for patients with psychiatric conditions within general hospital settings.

